# A Comparative Study on the Voxel Values in Alveolar Bones Acquired by MDCT and Newly Developed Dental Dual-Energy CBCT

**DOI:** 10.3390/s21227552

**Published:** 2021-11-13

**Authors:** Sungho Chang, Sang Chul Lee

**Affiliations:** Ray Co., Ltd., 12th Floor, 221, Pangyoyeok-ro, Bundang-Gu, Seongnam-si 13494, Korea; rayceo.lee@raymedical.co.kr

**Keywords:** dual-energy, cone beam, computed tomography, bone density

## Abstract

The purpose of this study was to analyze the effectiveness of newly developed dental dual-energy (DE) cone-beam computed tomography (CBCT) to compare both the voxel values in hard bone tissue of DE-CBCT and multidetector computed tomography (MDCT) images, collected in a clinical trial conducted at Seoul National University Dental Hospital. A software implemented as a scripted module of a three-dimensional (3D) slicer was developed to register the volume data from the MDCT space to DE-CBCT, locate the same 3D regions of interest (ROIs) in each image space, and extract the statistics of the ROIs. The mean values were paired and used as representative values of the ROIs. A scatter plot with the line of equality and Bland–Altman (BA) plot of difference for a pair of measured means were used for statistical analysis. Of the ROI pairs, 96% were within ±15% from the identity line, and more than 95% of the measured ROI pairs were within the limits of agreement of the 95% confidence intervals (CIs), with the CI of the limits in BA plots. The newly developed dental DE-CBCT showed a level of voxel value accuracy similar to that of MDCT.

## 1. Introduction

X-ray radiographs, such as panoramic and cephalometric images, and three-dimensional (3D) computed tomography (CT) or cone-beam computed tomography (CBCT) imaging are widely used in dentistry [1,2,3]. These X-ray-based modalities provide information on the morphology and internal structure of the dental hard tissues and alveolar bone. Images obtained using these modalities display relative degrees of solidity within the dental hard tissues. Whereas X-ray radiographs are projected images, in which the internal information of a subject is integrated along the beam trajectory, and thus is not adequate for assessing bone quality, CT or CBCT images represent the density of the subject and can be used to assess bone quality. The information on the density of the alveolar bones has a practical relevance, for example, in dental implant treatment planning, where local bone quality is known to be a strong predictor of successful implant osseointegration [4,5]. Various attempts were made to assess or analyze bone density using CBCT images [6,7,8,9,10,11,12,13]. Studies reported that the voxel values of CBCT images were generally not as accurate for representing bone density as those of general clinical CT images [14,15,16,17]. Dental CBCTs are inferior to clinical CTs in terms of tube current, peak tube voltage, detector acquisition rate, and signal collection efficiency. These physical limitations result in inaccurate voxel values and bone density assessments [18]. In the field of radiation therapy, some attempts to utilize deep learning techniques, such as the generative adversarial network (GAN), to correct CBCT artifacts, and to improve image quality of CBCT images were made [19,20]. A feasibility study was conducted to measure bone density directly and quantitatively from CBCT images using modified GAN [21].

To overcome the inaccuracy in voxel values of traditional dental CBCTs, we used dual-energy (DE) imaging technology and developed a new dental CBCT, RCT720. The principles and feasibility of this equipment were previously reported [22]. A feasibility clinical study was conducted at Seoul National University Dental Hospital (SNUDH) during the first half of 2019 and the results of that trial were published in [23]. A pivotal clinical trial investigating the application of DE-CBCT to assess jaw bone density and comparing this novel device with multidetector computed tomography (MDCT) was conducted at SNUDH in South Korea in 2020. Ray Co., Ltd. was a sponsor and received the subjects’ anonymized DICOM data. The purpose of this study was to analyze the effectiveness of DE-CBCT to compare the voxel values in the hard bone tissue of DE-CBCT and MDCT images, collected in the clinical trial.

## 2. Materials and Methods

### 2.1. Development of Dental DE-CBCT

We designed our DE-CBCT device, RCT720, to overcome the limitations of traditional dental imaging and provide assessments of jaw bone density, in addition to morphological information. A new kV-switching, high-voltage generator was developed and integrated into the existing dental CBCT system to acquire projected images at different kVp values. X-ray of low and high energy were alternately produced in pulsed mode with the kV-switching generator. The flat panel X-ray detector received the incident X-ray and then produced projection images alternatively at a frame rate of 50 Hz. The projection images at different kVp were collected separately, and CBCT images were reconstructed from the separate image data set, respectively. Using precalibrated parameters, the DE-CBCT images were calculated from the low-kVp CBCT images and the high-kVp CBCT images, voxel-by-voxel in the same coordinate. Our DE feature processed image data only in the image domain. A new calibration method was devised to estimate Hounsfield units (HUs) correctly from the slice images at different energies. The projection images were acquired with the hydroxyapatite (HA) phantoms of different densities inserted into the acrylic base at different kVp values, and the CT volumes were reconstructed separately. The voxels of the CT images at different kVp values were correlated point-by-point. We used the mean values of the phantom regions and the airy regions to estimate a line for density values and projected the correlated points of the regions onto the density line. The parameters for mapping the densities to HUs were found with known HU values of HA phantoms. Then, we calculated the new HUs from the CT volumes measured at different kVp values. The DE-CBCT image format was DICOM, and they could be viewed on a screen. We believe that, compared to conventional dental CBCT, the voxel values of the DE-CBCT images, HU, correctly reflected the density of the hard tissue, especially the jaw bone. References show that the HUs from CBCTs of various manufacturers were inconsistent with those of clinical CT. The final images were DICOM images, which could be displayed on any commercial DICOM 2D or 3D viewer. The device provided no additional DE-based imaging, such as material decomposition, DE subtraction, virtual monochromatic imaging, tissue characterization, or material separation, nor did it provide quantitative outputs from typical DE medical CT. Users or clinicians could select the region of interest (ROI) to assess the HU values on their image viewer and the bone quality classification of the voxels in the ROI provided in the DICOM viewer.

### 2.2. Conducted Clinical Trial

To evaluate the effectiveness and safety of DE-CBCT, we conducted a clinical trial after receiving the approval of the Institutional Review Board (IRB) of SNUDH (IRB No. CDE19006) in South Korea. The aim of the trial was to assess jaw bone density and compare these values with MDCT, an established standard for assessment of bone density. The study was an open-label clinical trial evaluating the effectiveness and accuracy of DE-CBCT for assessing BD in the jaw bones. A total of 34 subjects were enrolled in this single-center trial (17 each for the maxilla and mandible). Patients were recruited based on three criteria: (1) aged 19–85 years; (2) required CT imaging for dental diagnosis, especially implant treatment planning; and (3) voluntarily signed a written consent. The following patients were excluded: those who were pregnant or lactating, received a CT scan the day before or on the same day, were unable to read or write, or had cognitive impairment. Other cases were excluded if the researcher determined that participation in the clinical trial was inappropriate, as it could affect the clinical trial results, or for ethical reasons. Each study subject received both the investigational DE-CBCT and the standard-of-care MDCT scan, which served as the control. This study did not include a randomization into control and treatment groups. The investigational device was RCT720, a newly developed dental DE-CBCT scanner, and the control device was an MDCT scanner (Siemens SOMATOM Definition Edge), which was already installed in the SNUDH.

### 2.3. Software Development to Extract Information of the ROIs

The DICOM data from different modalities had different geometric characteristics, such as resolution and pixel pitches or spacing. The resolution of the DE-CBCT image was 334 × 334, pixel pitches were 300 μm and 300 μm, and slice thickness was 300 μm. Images acquired with the MDCT had 512 × 512 resolution, 332 μm × 332 μm pixel spacing, and slice thickness of 750 μm. Although the CT images of each device were acquired with the same patient, it was difficult to directly compare images taken with different equipment at different times, because the patient’s posture or internal structure might be different. To quantitatively compare the measured volume data, the image analysis software must be able to register two similar volumes in the same space. It must also be able to place the same ROI on each image space with a selection of the ROI and extract the statistics from the selected ROI.

**Figure 1:**
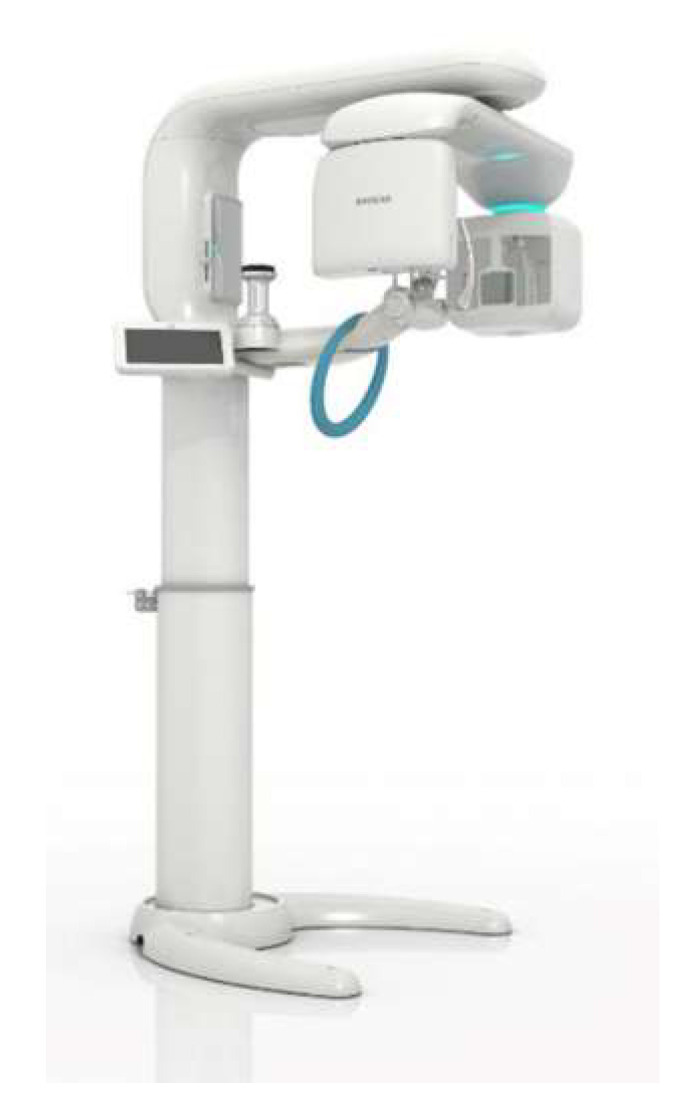
Rendering view of the RCT720.

**Figure 2:**
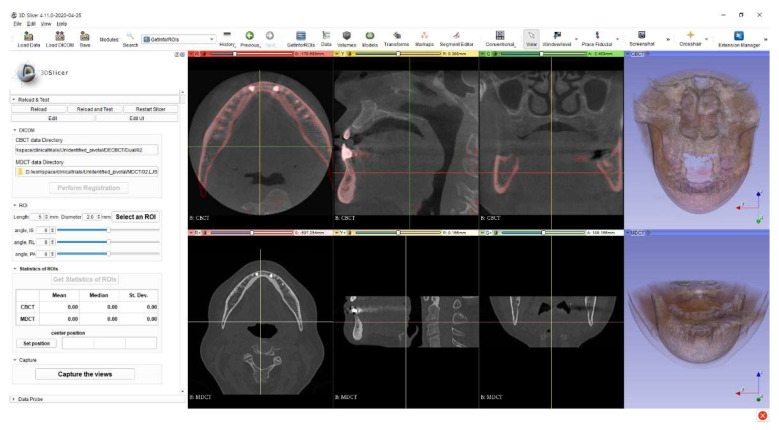
A screenshot of the developed 3D slicer module. Registered multi-detector computed tomography (MDCT) volume was represented as the red shade upon dual energy (DE) cone beam computed tomography (CBCT) volume.

Commercial software did not meet our requirements, and it was hard to develop 3D image analysis software from scratch. Thus, we decided to develop software to evaluate the statistics of selected ROIs as a scripted module of 3D Slicer [24], which is a well-established, easily extensible, and open-source software. The developed software had a customized graphical user interface on the left side, with two rows of multiplanar reconstruction (MPR) views and 3D rendering view placed in the image display area. The DICOM data of each measurement by each modality were displayed through MPR views and 3D rendering view, respectively, as shown in Figure 1. The registration feature was implemented with SimpleElastix [25]. The transformation matrix of registration from MDCT to DE-CBCT was inverted and used to transform the selected ROI on the image space of DE-CBCT to that of MDCT. We used the existing 3D slicer’s modules to select an ROI and extract the statistics from the ROIs. We used the ROIs with a cylindrical shape, and the geometric parameters of the ROI such as height, diameter, and rotation angles about three axes could be adjusted to be fitted in the bone tissue. The heights of the ROIs were adjusted from 4.0 mm to 5.0 mm, and their diameters were between 1.0 mm and 1.4 mm. The three rotation angles of ROIs could be changed in the range of ±90°. Figure 2 shows the screenshot after registration with the loaded MDCT and DE-CBCT images. The views under the red, yellow, and green bars are the axial, sagittal, and coronal views, respectively. The red color represents the bone region of MDCT volume into the DE-CBCT space.

**Figure 3:**
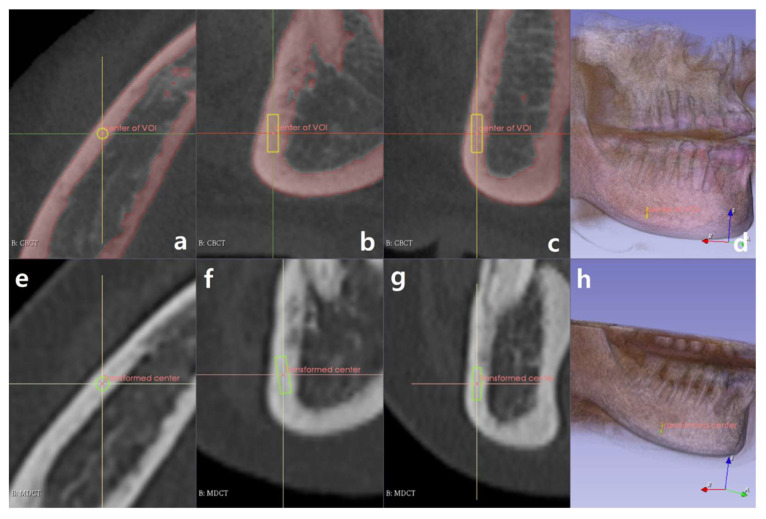
MPR views of DE CBCT and MDCT. Yellow perimeters and green perimeters indicate the selected region-of-interest (ROI) and transformed ROI, respectively: (**a**) axial view, (**b**) sagittal view, (**c**) coronal view, (**d**) 3D volume rendering of the DE CBCT image, (**e**) axial view, (**f**) sagittal view, (**g**) coronal view, and (**h**) 3D volume rendering of the MDCT image.

Figure 3 displays the selected ROI and transformed ROI with loaded DICOM images and 3D-rendered images. The boundaries of the ROI user selected are shown in yellow in Figure 3a–d, and the boundaries of the transformed ROI are displayed in green in Figure 3e–h.

### 2.4. Criteria for Selecting ROIs

**Figure 4:**
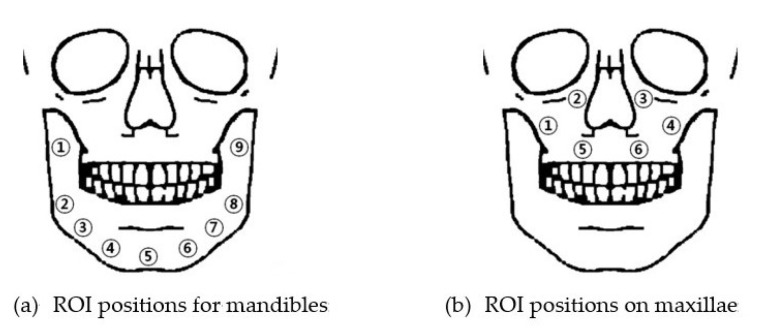
Reference positions of ROIs (**a**) on mandibles and (**b**) on maxillae.

Because the shapes and thicknesses of the mandibles and maxillae of the patients were different from each other, it was not possible to locate each ROI to the same positions in each mandible and maxilla. Thus, determining the reference positions and placing each ROI as close to each reference position as possible was the optimal solution. The number of reference positions in the mandible and maxilla were 9 and 6, respectively, and the positions are shown in Figure 4. The size and angle of the ROIs were adjusted so that both the selected and the transformed ROI contained as much cortical bone as possible and avoided defects in the bone tissues. The mean values of each ROI were used as representative values. The mean values of the selected and transformed ROIs were used as paired data for statistical analysis. The registration of the images of 3 of the 17 subjects in the maxillary and mandibular cases, respectively, failed, and thus were excluded from the ROI analysis. A total of 126 ROIs were from the mandible case subjects and 84 were from maxilla cases.

### 2.5. Statistical Analysis

To show how close the CT values of our DE-CBCT were to those of MDCT, we plotted the data pairs as a scatter plot with an identity line. If the pairs of the measured values were the same, the scattered dots lied on the identity line. The vertical distance from the identity line to a plotted point represented the deviation between the measured pair. The Bland–Altman (BA) plot [26] is a very useful tool for comparing two measurements, as it assesses the relationship between the differences and the magnitude of paired measurements, bias (as mean difference), and limits of agreement between the two methods or conditions. If the differences are approximately normally distributed, then approximately 95% of the differences should be within these limits. The BA plot was based on the assumption of normality, and the data could be considered as normally distributed; thus, we performed the normality test before analyzing the paired measurements with the BA plot. To determine if the distribution was normal, normality tests should be used, because in some cases, normality could not be determined simply by observing the histogram plot. Quantile–quantile (QQ) plots were drawn as another test to visually check normality. The Shapiro–Wilk (SW) test was used to assess the normality of the data [27] using the software R [28]. Table 1 presents the results of the SW test. The *p* value of the SW test for each ROI group was higher than the widely used significance level of 0.01.

## 3. Results

### 3.1. Scatter Plot over the Identity Line

**Figure 5:**
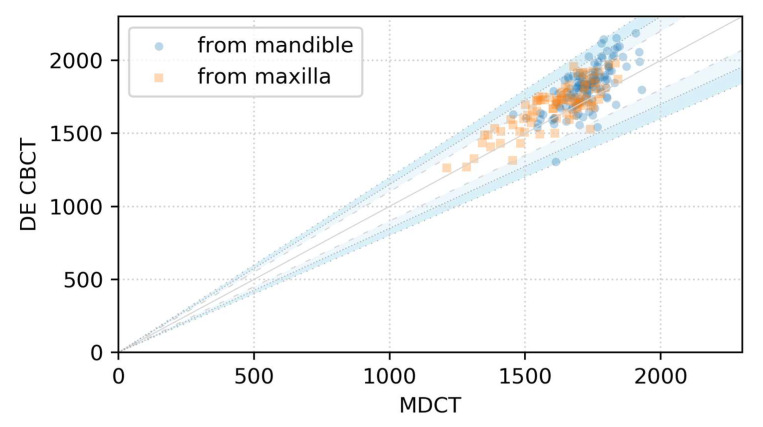
Scatter plot with the identity line and deviation ranges.

Figure 5 shows the scatter plot with the identity line and deviation ranges. The solid line is the identity line, dashed lines represent the range of ±10% deviation from the identity line, dotted lines represent the range of ±15% deviation from the identity line, and loosely dotted lines represent the range of ±20% deviation from the identity line. The number and percentage of ROIs within each deviation range are given in Table 2. Of 210 ROIs, 97.1% lie within the ±15% range, and 2.9% are in the range between ±15% and 20%.

### 3.2. BA Plot Analysis

**Figure 6:**
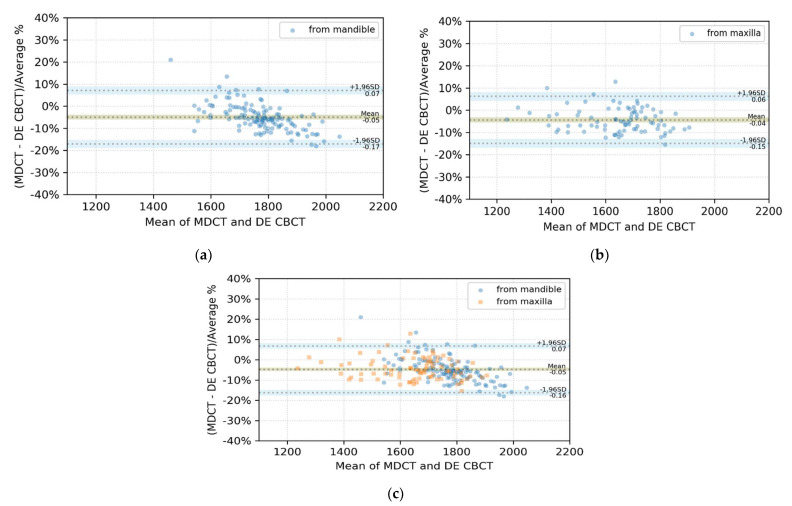
BA plots of the differences between paired ROI means of MDCT and DE-CBCT, expressed as percentages of the values on the axis ((MDCT-DE-CBCT)/average %)) vs. the mean of each data pair (**a**) of mandibles, (**b**) of maxillae, and (**c**) of mandibles and maxillae. The shaded areas represented confidence interval limits for the mean and limits of agreement.

The BA plots of the differences of the measured pairs from mandibles, maxillae (separately) and mandibles and maxillae together, with their limits of agreement, are shown in Figure 6a–c, respectively. The shaded areas in each plot represent the CI limits for the mean and agreement limits (ALs). Two of the 126 data points (1.6%) lay outside the 95% CI and CI limits for ALs, as seen in Figure 6a. Of the 84 data points, 82 (97.6%) lied inside the 95% CI and CI limits for ALs, as seen in Figure 6b. However, 7 of the 210 dots (3.3%) were outside the 95% CI and CI limits for ALs, as seen in Figure 6c.

## 4. Discussion

The results of this study show that there is no statistically significant difference between the voxel values measured by DE-CBCT and MDCT. In comparing the voxel values from two different modalities, selecting ROIs is the most important and fundamental procedure. Because the shape and thickness of the maxilla and mandible vary from patient to patient, it is impossible to find an ROI in the same location in all patients. Although the anatomical structures recorded in the CT images acquired with each modality have different sizes, shapes, and angulations, the images from each modality should be registered. The position, height, diameter, and angle of rotation of the ROIs are positioned close to their respective reference positions and adjusted to include as much tibial tissue as possible and to exclude defect sites as much as possible.

As shown in Figure 5 and Table 2, nearly 80% of the total points showed a deviation within ±10% from the identity line. The points of deviation >10% and <15% comprised about 18% of the total, and the sum of the two cases was greater than 96%. With the combined data of maxillae and mandibles, <3% of points lied over the ±15% deviation from the identity line, and all points deviated less than 20% from the line of equality. ROI values from DE-CBCT were close enough to those of MDCT, and it can be said that they have the same level of accuracy as MDCT, compared with conventional CBCT.

To apply BA plots to the differences of the measured ROI values, each group of differences should follow a normal distribution. The *p* values of the SW test as a normality test on each different data group were >0.01, and each data group could be interpreted as following a normal distribution.

All three plots in Figure 6 had negative biases and means of differences. This means that the ROI values of DE-CBCT tended to be larger than those of MDCT. As shown in Figure 6a,b, 2 of the 210 points were outside the limits of agreement and the CI of the limits, respectively. We drew a BA plot as in Figure 6c. As shown in Figure 6c, six points lay outside the intervals with the combined ROI data, whereas only two points were outside the intervals, as shown in Figure 6a,b, respectively. This resulted from the change in the mean and standard deviation of the differences as the two groups are merged, and these changes affected the limits of agreement and their CI. The biases in each ROI group and the gaps between each bias and each limit of agreement are given in Table 3. The bias and the gap between the bias and the AL of the ROI from the mandibles were higher than those from the maxillae. Because 98.41%, 97.62%, and 97.14% of the ROIs for the mandibular, maxillary, and combined data, respectively, were within the range of the 95% CI and the CI of the AL, the voxel values of DE-CBCT and MDCT showed good agreement with 95% CI in each case.

RCT720 took approximately 30 s from the start of the patient scan to image reconstruction and provided resultant images. This was expected to be faster than deep learning techniques [20,21], which perform massive computation through complex neural networks after patient scan and image reconstruction. Furthermore, we expect that it would be more convenient and familiar to assess bone quality with the voxel values of CBCT images than with bone density values [21].

It is difficult to quantitatively evaluate bone tissue using conventional dental CBCTs [14,15,16,17]. The accuracy of RCT720’s voxel values of bone tissues might be helpful to establish a treatment plan in daily practice. However, RCT720 was developed only by focusing on the voxel value accuracy; therefore, it cannot be applied clinically as conventional clinical DE CTs.

Our device RCT720 was permitted to be freely sold in domestic and overseas markets on 31 August 2021 in Korea based on the results of the pivotal clinical study. However, approval from the relevant country must be obtained to export this device to other countries.

## 5. Conclusions

We developed a novel dental DE-CBCT RCT720 and a software to extract ROI. ROIs were extracted from the registered images at the same location in each image space. Pairs of ROI values were compared using Bland–Altman plots. The newly developed RCT720 showed a level of voxel value accuracy close to that of MDCT.

## 6. Patents

Chang, S. and Lee, S.C. (2019). Method of obtaining calibration parameters for dual-energy cone-beam computed tomography images (10–2070373–0000). Korean Intellectual Property Office.

## Figures and Tables

**Table 1:** *p* values of Shapiro–Wilk test on each ROI group.

ROI Group	*p* Value
Mandible	0.095
Maxilla	0.213
Mandible and maxilla	0.047

**Table 2:** Deviation ranges and region of interest (ROI) numbers and percentages within each deviation range.

	Deviation from the Identity Line		
ROI Group	Within ±10%	Between 10% and 15%	Greater Than 15%
Mandible	97 ROIs; 77.0%	24 ROIs; 19.0%	5 ROIs; 4.0%
Maxilla	68 ROIs; 81.0%	15 ROIs; 17.9%	1 ROI; 1.2%
Mandible and maxilla	165 ROIs; 78.6%	39 ROIs; 18.5%	6 ROIs; 2.9%

**Table 3:** Bias, gap between the bias and the agreement limit (AL), and the confidence interval (CI) of the AL of each ROI group.

ROI Group	Bias	Gap between the Bias and the AL	CI of AL
Mandible	−4.94%	12.09%	3.75%
Maxilla	−4.34%	10.57%	4.01%
Mandible and maxilla	−4.70%	11.39%	2.76%

## Data Availability

Restrictions apply to the availability of these data. Data were obtained from SNUDH and are available with the permission of SNUDH IRB.
